# On the Drainage of the Metropolis by a Continuous Water-Flow

**Published:** 1857-04

**Authors:** George E. Nicholas

**Affiliations:** District Medical Officer, and Medical Officer of Health for Wandsworth; formerly Assistant-Surgeon, *R.N*.


### ON THE DRAINAGE OF THE METROPOLIS BY A CONTINUOUS WATER FLOW.*

By GEORGE E. NICHOLAS, District Medical Officer, and Medical Officer of Health for Wandsworth; formerly Assistant-Surgeon, *R.N.*

THE question of the drainage of the metropolis has now been under consideration for twelve months by a Board expressly constituted for the purpose of its solution, without advancing a single step towards the accomplishment of that object. The cause of this shortcoming is manifestly attributable, not to any deficiency in plain practical common sense or in engineering skill, but to the absence of that essential in their deliberations upon a purely sanitary question—a knowledge of sanitary science, and to a consequent absence of data upon which either a correct system could be founded by the engineer, or a correct judgment of such or any system could be arrived at by its members. It is evident, and to be regretted, that this hiatus in their counsels was not recognised; otherwise it would have been at once apparent, to the saving of much valuable time (and, it may be added, to the verification of the Roman proverb, "ne sutor ultra crepidam"), that the operations of the engineer could only be subservient to certain purposes; which purposes, being sanitary ones, it would devolve upon those whose scientific attainments entitle them to be the exponents of the laws relating to the promotion of health and the prevention of disease, and not the engineer or themselves, to determine. In other words, it would be necessary—1. To determine the conditions which sanitary science demands for the accomplishment of the object to which drainage is applied, namely, the preservation of the public health; and 2. To project the skill of the engineer to the complete fulfilment of those conditions.

However much the present tendency of popular opinion may be to confuse the subject by considerations of false economy, the question is undoubtedly a sanitary one, and is embraced in the above two propositions. If the before named conditions be satisfactorily determined and defined, there can be no doubt that more than sufficient engineering skill† exists for their fulfilment; but, unless such requirements be first fully entertained, it is equally doubtless that the operations of the engineer will form but a grand mass of fruitless ingenuity.

---

* The substance of the following remarks was addressed to the Metropolitan Association of Medical Officers of Health, at a meeting held on Jan. 28, 1857.

† Sir Wm. Cubitt and Mr. Stephenson, in their Report of the 11th Dec. 1854, state: "No part of engineering science has been more industriously investigated than the laws which govern the flow of water in pipes and open channels", etc.

The initiative consideration of the subject peculiarly belongs to the province of the medical officer of health. I trust I need offer no excuse, therefore, in soliciting earnest attention to the subject; inasmuch as it would appear but inconsistent with the objects of this Association, that its members—the elected guardians of the public health—should remain inactive, in a matter which so deeply concerns their public duties.

Apart from these considerations, the subject is most worthy of attention for its own merits, the importance of which cannot be overestimated; for of all the causes operating to the increase of the Bills of Mortality, within the official cognisance of the medical officer of health, imperfect drainage is one of the most, if not the most, potent; while it is at the same time the most easily recognised, the most easy (as I believe) to be remedied, and, when remedied, the most productive of beneficial results. For how comparatively trifling, and slow, and gradual, must necessarily be the process which can ever be expected to be realised in the alleviation of other sanitary evils, such as the sale of adulterated and unwholesome food, the dampness, uncleanness, bad ventilation, and especially overcrowding of houses, etc., in comparison with the great and immediate beneficial results to be derived from a perfect system of drainage, the establishment and subsequent working of which can be effected without being obstructed by the trammels of private interest, and that vast amount of ignorance and prejudice, which stand in the way of the removal of other sanitary evils, such as I have just mentioned. The importance of the subject need not be further urged: it admits of no denial.

In the consideration of this, as of every other subject, correct conclusions can only be educed from true premises. A sound foundation is necessary for a firm superstructure. With this view, I take for a basis the well established fact, as evidenced in the returns of the Registrar-General, and confirmed by all experience; viz., that every population living in an atmosphere rendered impure by those gases which result from the retention, and consequent decomposition, of the excreta of the community, beneath or in the vicinity of its habitations (as in cesspools, or imperfect sewers or drains), becomes subject to a lowered vitality, and prone to the reception of disease, especially that of an epidemic or contagious nature. Therefore,—

1. The existence of a means by which the removal of the ordure of the metropolis may be accomplished, is a paramount necessity for the health of its inhabitants.

2. This removal must be immediate, in order to preclude decomposition and its resulting evils.

3. The means of this removal must be inoffensive in itself.

4. In the adaptation of this means for the prompt and inoffensive defæcation of the town is comprised the entire object of drainage.

These are the data which it has appeared to me essentially necessary to keep prominently in view, in any attempt at arriving at a plain, true, and practical exposition of the subject. But, before proceeding to deduce conclusions from them, it will be well first to see what assistance history furnishes on the subject.

The earliest reference to the subject is found in the Mosaic laws, relating to the observance of cleanliness by the army when encamped; it was here ordained that each individual should go forth without the camp, and, with a paddle adapted to his weapon for the purpose, inter his own ordure.*

That a somewhat similar custom was almost universal at the time of Herodotus, may be inferred from the circumstance of that author mentioning, as one of the peculiarities of the habits of the Egyptians, by which they differed from other nations, that they "discharged their excrements at home."† Here it may be remarked, that the present cesspool system, which prevails generally in modern civilised life, but which has of late been so wisely interdicted in this metropolis, would appear to be but a modification of the foregoing custom, localised by necessities resulting from a town life.

The Chinese, who have retained most of their ancient customs to the present time, furnish the next departure from the *outdoor* custom. Regardless of sanitary considerations, they store their ordure in tubs (portable cesspools), for the purpose of selling it to the rustics, who come into town every morning to collect and buy it at so much the pound. The laws of the State forbid that any should be thrown away; for, in consequence of the scarcity of cattle, it becomes very valuable as manure. They are the most industrious of agriculturists.

The Athenians, with whom civilisation had greatly advanced in the time of Solon, do not appear to have possessed any works of drainage; otherwise reference to them would doubtless be discoverable amongst the many social laws of that great lawgiver.

It was reserved for the wisdom of the Romans to emancipate man from habits which so nearly allied him to the animal creation, by the projection of a system of drainage, which, whether considered in a sanitary or other point of view, remains unrivalled at the present day; and which, of all their grandest works, forms perhaps the most prominent monument of their

---

* Deuteronomy xxiii, 12 and 13.

† Herodotus, book ii.

refinement and civilisation. The drainage of Rome, which is well described by Pliny,* consisted of a system of sewers (cloacæ), which received the surface water and dirt of the streets, as well as the ordure† from the houses of the whole city; the communications between the houses and sewers being effected by earthen or wooden pipes. These sewers opened into one main (cloaca maxima), which was fifteen feet wide and thirty feet high, and was navigable by boats.‡ Some portions of it are still in existence. The most remarkable feature, however, of the system, and which is most instructive, is the abundant supply of water with which it was furnished, as evidenced by the large number of aqueducts, the universal use of the bath, the numerous public and private fountains, fishponds, and artificial lakes, and the seven streams§ which fed the main sewer, the rapidity of whose current equalled that of the Tiber, into which it flowed.

We look in vain for any such efficient system in modern times. The drainage of London most resembles it, but is, as will be seen on examination, defective in the very element which essentially gave to the drainage of Rome its excellence—an abundant and *continuous* flow of water.

The removal of the ordure of this metropolis has hitherto been effected partly by horse and cart, assisted by manual labour, and partly by waterflow. The former, inasmuch as it was most offensive in itself, and involved the use of cesspools, thereby giving rise to all those evils which it is necessary to avoid, has been most wisely interdicted. The latter remains in the form of the present drainage, which, as regards its extent in connexion with its results, is remarkable not only for its inefficiency but for the production of much mischief. The cause of this inefficiency arises not from any defect in the principles upon which it is based, but on account of the inefficiency of its most essential element—the vehicle, the motive power, the water—which takes the place of that labour which man, in his primitive state, must perform, and for the lessening of which drainage was invented by him. In consequence of this defect, all those matters—the dilution of, and immediate and inoffensive removal of which it is intended to effect—are arrested, accumulate, and, suffering decomposition, give off those noxious gases, which it is the custom to confine and concentrate by "trapping" and other mechanical contrivances;

---

* Pliny, Opus omnium Maximum, etc., lib. xxxvi, cap. xv.

† Ulpianus (Domitius).

‡ Dion Cassius.

§ On the accession of Augustus, Marcus Agrippa restored the sewers, then out of repair, and caused seven streams to flow into the main sewer. Dionysius Halicarnassus.

but which, by the laws of gaseous diffusion, somewhere or other find an exit, and there exert their morbific influences. This great defect has illustrative proof in the fact that the sewers have to be periodically relieved of their accumulations by large flushings of water, at great expense and great injury to them, and ultimately by manual labour—a practice which every medical officer of health will confirm me in stating to be morally and physically wrong, as repugnant to human nature, injurious to health, and sometimes destructive to life.*

A consideration of the foregoing data and evidence adduced leads to the conclusion that the present drainage, although possessing neither uniformity, regularity, nor efficiency, and although an extension of it in its present defective state (as contemplated by the engineer of the Metropolitan Board of Works) would but multiply its existing evils in proportion to such extension, nevertheless contains the principles of that system which it would be most desirable to pursue to its ultimate perfection; for there is ample experience to prove that the most simple, convenient, and inoffensive, as well as economical and efficient means which could be possibly devised for the accomplishment of the object of drainage, is afforded by a flow of water by gravitation, which serves at once for the dilution of offensive liquids, the absorption of gases, as the vehicle of the solid matters, and as the motive power for the removal of the whole. But this perfection can only be ensured by making provision for a continuous flow of water throughout the whole system, beginning at its periphery, consequently from each house. This supply of water should be independent of rain, the refuse of that used for domestic purposes, or any other accidental sources; it should be entirely removed from the control, and consequently from the carelessness or neglect of individual members of the community; it should be furnished in such quantity as to keep the sewers as full as practicable, in order to ensure the prevention of the accumulation of gases which may occur in sparingly filled sewers, and the absorption of any as soon as formed, by continuous fresh volumes of water, and also to diminish that great amount of wear and tear which arises from forcible and interrupted flushings. The sewers should be impermeable by liquids or gaseous fluids; and it may be assumed that very much smaller ones than those now in use might be advantageously employed if the rapidity of re-

* Many instances have occurred of the death of men engaged in this disgusting labour. In 1849, a party of men, and a medical gentleman (Mr. Wells) who went to their assistance, lost their lives in the Warwick Street sewer. Much sickness has also resulted from such occupation, of which the public are mostly unaware.

moval, which a continuous flow of water would ensure, be taken into account. It would be matter for serious consideration how this large supply of water is to be furnished, whether by the Thames and its tributary rivers, by artesian borings, or by the construction of reservoirs of sufficient size capable of being filled at high water, or by all of these. The last mentioned would appear to be sufficiently practicable, except for those portions of the metropolis whose elevation is too great. But in the absence of these, and inasmuch as reasonable fears have been entertained that the removal of so large a body of water as that now required from the Thames and its tributaries will before long become injurious to the natural constitution of the river, and seeing that such removal is rapidly increasing, the proposition of bringing by aqueduct from the sea an unlimited supply is so reasonable and practicable that it should be at once embraced. Such a supply of salt water would at once serve for the purposes of drainage, street washings, public and private baths, fountains, and lakes; and, in short, for very many other purposes for which filtered river water is now employed; while it would be the means (and this is most important) of causing an immense saving in the expenditure of that vital necessity the drinking-water of the people, and correspondingly diminished withdrawal from the Thames and its tributary streams. To these advantages may be added that which such a supply would possess in the extinction of accidental fire. This proposition has been objected to on the score of the increased expense which it would entail. It will be found on examination, however, to contain a vast economy. It has been confidently stated that such a supply could be furnished at the low rate of one penny in the pound upon all rentals. The present water rate is about ten times this amount. The difference between the two rates, relative to the amount of filtered river water which would be superseded by salt water, would be the amount saved upon the whole water rate.

With reference to the point of the outlet of the sewerage, numerous propositions have been made, according to the various intentions entertained as to the ultimate utilisation or loss of the sewerage. In dealing with this very important part of the subject, it is necessary to remember that as the object of drainage is simply, and without any modification, a purely sanitary one, so the means for the accomplishment of that object should not be hampered or interfered with by any abstract theory or plan of a secondary nature; that, however desirable it may be to conjoin with it a means of restoring the sewerage of the town to the fields from whence it originally came, that the town may derive a pecuniary benefit by the transaction; this should not be tried unless it can be done without the least obstruction to, or interference with, the primary object in view, which is not a financial but a sanitary one.

The most practicable of the above propositions are—1. To convey the sewerage to certain works for the purpose of deodorising it, and converting it into manure. 2. To convey it immediately to the fields, for the purpose of manuring them by irrigation. 3. To convey it to the sea. This would include the proposition to carry it so far down the river as to prevent the possibility of reflux, which would be virtually taking it to the sea.

1. In examining the various plans of deodorisation which have been brought forward, the first great objection which presents itself, and which appears insuperable (premising that drainage by water flow is the only one from which efficiency can be anticipated), is the obstructiveness to the object of drainage which they contain; for it is admitted that the value of town ordure for manufacturing manure by deodorisation is diminished in proportion to the amount of water with which it is diluted—a fact which is in opposition to the drainage by waterflow.

Passing by this objection, let us inquire what conditions chemistry is required to fulfil, and whether that science has the power to fulfil them. Sanitary considerations demand that the sewerage shall not only be deodorised but disinfected also, which is another and very different thing; for infection may exist, although inappreciable to the senses. Deodorisation and disinfection have been so confounded by writers, even of the present day, that these terms are frequently used synonymously; their action has also been equally confused. Deodorisation, which represents the power of destroying offensive odours, and of arresting the putrefactive process, may be readily effected by many chemical agents, particularly the alkaline chlorides; but there is no evidence to shew that any such substances have the power of destroying those newly formed products of putrefaction which are presumed to be either the direct or indirect, the active or passive agents in the propagation and promotion of disease. On the contrary, there is much incontroverted evidence from experiments to prove the reverse. Thus: vaccine lymph is found to succeed, when mixed with an equal quantity of solution of chloride of soda. Chlorine was employed some years ago at the Small-pox Hospital for the arrest of erysipelas; all offensive smell was suppressed, but not so the extension of the disease. During the cholera at Moscow it was found that, at the time when the Cholera Hospital was filled with clouds of chlorine, then it was that the greatest number of attendants were attacked. In fact, the only real disinfectant known is heat.*

With all due recognition of the great and increasing powers of chemistry, I cannot help expressing my conviction that, at the present time, that science is not sufficiently advanced to undertake the fulfilment of such conditions as sanitary science demands in any proposed works of deodorisation.

2. By the plan of irrigation, the greatest financial return would be undoubtedly realised. It would also appear to be supported by the solution of a natural problem—the direct restoration of that fertility to the soil of which it has been deprived by cultivation, thus completing the link in the true physiological circle between the animal and vegetable kingdoms. But it is a matter for grave consideration whether the constant exposure of so large a body of offensive matter† over so large a surface as it must occupy, would not develope far greater evils than the partial, or even the entire loss of value which would accrue from the plan of carrying it to the sea.

3. Carrying ordure to the sea, it is generally thought, would be tantamount to throwing it away. There is good reason, however, for believing that, in a financial point of view, it is not lost at the present time; for the fishermen say that it is returned to the alderman's table in the form of fat wholesome fish. It is a certainty that fish congregate at the mouths of rivers (which cannot, at the mouth of the Thames, be to obtain fresh water as imagined), and it is reasonably supposed that the *debris* of organic tissues present in the sewerage furnish a great amount of the food of fish found on inhabited coasts. This consideration would be more important and noticeable, should Government pay that attention to its fisheries which is found to yield so abundant a return in America and elsewhere.

Loss of life and loss of sewerage attend the present and the proposed systems; but let the only available means be adopted, viz., an abundant and continuous water-flow, for the dilution and immediate removal of all sewerage, and the greater of these losses will be as immediately prevented. Until chemistry, which now appears inclined to lend herself to the sewerage market, shall be able to step in, and, assuming an unmistakeable position, save by her appliances both life and sewerage, it is evident that either the sewerage must be economised at the cost of health and life, or health and life must be preserved at the expense of the sewerage. Of these two evils shall we not choose the lesser?

---

* In a pamphlet on the Drainage and Sewerage of London, etc., by Dr. Copland, this, the most important question in reference to works of deodorisation, is summarily disposed of by means of a little chloride of lime. (p. 24.)

† The daily amount of the excreta of London exceeds 1100 tons.

